# Longitudinal relationship between internet self-control and problematic internet use among Chinese adolescents: mediating role of meaning in life

**DOI:** 10.3389/fpsyt.2023.1258673

**Published:** 2023-12-07

**Authors:** Weijun Wang, Jianmei Ye, Yimeng Zhu, Dawei Huang, Xin Zhao

**Affiliations:** ^1^Key Laboratory of Adolescent Cyberpsychology and Behavior, Ministry of Education, Wuhan, Hubei Province, China; ^2^School of Psychology, Central China Normal University, Wuhan, Hubei Province, China; ^3^Institute of Digital Commerce, Wuhan Technology and Business University, Wuhan, China; ^4^Key Laboratory of Human Development and Mental Health of Hubei Province, Wuhan, China; ^5^Information School, The University of Sheffield, Sheffield, United Kingdom

**Keywords:** problematic internet use, internet self-control, meaning in life, cross-lagged panel model, longitudinal mediation model, adolescents

## Abstract

**Introduction:**

While studies indicate that high self-control may serve as a safeguard against problematic internet use, there’s evidence suggesting that problematic internet use can, in turn, diminish self-control. This study aimed to elucidate the longitudinal interplay between internet self-control and problematic internet use in adolescents, employing cross-lagged panel modeling. Furthermore, drawing from a positive psychology perspective, we examined the potential role of ‘meaning in life’ as a protective mediator within this longitudinal relationship. We then constructed a mediation model to explore protective factors against problematic internet use.

**Methods:**

Through a questionnaire, we tracked 659 adolescents (331 males and 328 females; mean age=13.61) in a longitudinal design across two time points, spaced at five-month intervals, to assess their internet self-control, problematic internet use, and meaning in life.

**Results:**

Results of the cross-lagged panel models showed that: Internet self-control had a significant negative impact on problematic internet use after five months (*β* = −0.094, *p* < 0.01). Conversely, problematic internet use had a significant negative impact on internet self-control after five months (*β* = −0.099, *p* < 0.05). Results from the longitudinal mediation model showed that: Meaning in life mediated the effect of internet self-control on problematic internet use after five months (*β_internet self-control(T1)-meaning in life(T2)_* = 0.142, *p* < 0.01; *β_meaning in life(T1)-problematic internet use (T2)_* = −0.075, *p* < 0.05).

**Conclusion:**

Our study uncovers a reciprocal predictive relationship between internet self-control and problematic internet use, while highlighting the mediating role of meaning in life within this relationship. These findings suggest that fostering internet self-control and cultivating a sense of meaning in life among adolescents can serve as effective prevention and intervention strategies for addressing the issue of problematic internet use.

## 1 Introduction

According to the latest report from the “China Statistical Yearbook 2021,” the number of Chinese adolescents, aged 7–18, engaging as internet users has surged to 191 million, resulting in a staggering internet penetration rate of 96.8% among minors (1). Minors use the Internet for an average of more than 5 h on holidays (2). According to the standard criteria for identifying behavioral addictions, excessive involvement in any type of activity (e.g., compulsive buying, binge eating, excessive work involvement, and excessive internet use) may be classified as a psychiatric disorder (3). Excessive, compulsive, and uncontrollable use of the Internet is referred to as problematic Internet use (PIU, also known as internet addiction). PIU may lead to functional impairment in daily activities (4, 5), adversely affecting the overall well-being and societal functioning of adolescents (6, 7). PIU has emerged as a significant mental health concern (8), particularly among adolescents (9). Unregulated internet usage among adolescents has been found to have detrimental effects on their self-adaptation and social skills (10). Furthermore, it is associated with an elevated risk of social anxiety, depression, and loneliness (11). Additionally, excessive internet use has been linked to higher levels of academic procrastination (12), poorer academic performance, and reduced academic achievement among adolescents (13). Research has indicated a considerable prevalence of PIU among Chinese adolescents, estimated at 7.7% (14). These findings underscore the need for further research exploring the mental health correlates and potential interventions for PIU in adolescents in China, who appear to be particularly vulnerable.

Owing to the detrimental effects of PIU on the psychological development of adolescents, prior research has endeavored to explore associated risk and protective factors, such as, impulsivity, codependency (15), meaning in life, and self-esteem (16), self-efficacy and self-control (17). Notably, self-control has emerged as a prominent factor, with empirical evidence demonstrating its associations with the severity of PIU among adolescents (18–20). However, bidirectional effects have been noted, with PIU also inversely predicting adolescent self-control (21). At present, the temporal precedence and mediating pathways underlying the relationship between adolescent self-control and PIU remain elusive. Understanding causal pathways and underlying mechanisms linking self-control and PIU will inform targeted preventative interventions. Therefore, the objective of our study is to elucidate the associations between self-control and PIU in the online context among adolescents, while also exploring potential mediators within this relationship.

### 1.1 Internet self-control and problematic internet use

According to the basic psychological needs theory (22), the failure to satisfy an individual’s three fundamental psychological needs of autonomy, competence, and belonging can have a negative impact on them. Wu et al. (23) found that adolescents often develop an addiction to online games as a result of their unmet needs for autonomy and a sense of competence in real life. Throughout the developmental process of adolescents, various needs arise, and when these needs are inadequately fulfilled, a process of “pathological compensation” occurs (24). The Compensatory internet use (CITU) posits that individuals might turn to online activities as a way to cope with negative life situations and alleviate distressing feelings (25). In this process, adolescents may engage in activities such as online gaming, which can provide a readily accessible sense of accomplishment as a form of compensation. Consequently, the capacity for self-control and regulation becomes a significant determinant in the occurrence of PIU (26).

Self-control refers to the ability to suppress immediate impulses and regulate one’s behavior (17), while internet self-control specifically pertains to an individual’s capacity to regulate their behavior on the internet (27). Current research suggests that high levels of self-control serve as a protective factor against PIU in adolescents (20, 28). Individuals with greater self-control are shown to effectively regulate their internet use, resisting the allure of stimulating elements in the virtual online world. They tend to exhibit rational control over their online activities and are less prone to excessive immersion in the pleasurable aspects offered by the online environment (29). Conversely, individuals with lower levels of self-control are more susceptible to becoming absorbed in the online world. They often lack effective management and regulation of negative emotions, turning to PIU as an escape from real-life issues (26). Furthermore, individuals with lower levels of self-control are more inclined to depend on the internet to meet their emotional needs. This indulgence in utilizing the internet for personal gratification can potentially exacerbate the progression of PIU (20).

According to basic psychological needs theory, individuals with high levels of self-control often exhibit greater autonomy and possess the ability to independently manage their online behavior. They can effectively regulate their internet usage time and activities, thereby minimizing the risk of excessive immersion and addiction (30). In contrast, individuals with low levels of self-control, upon engaging with the internet, tend to find that the diversity and convenience of the online world expeditiously fulfill their basic needs. As the internet’s capacity to meet individuals’ psychological needs increases, they become more susceptible to excessive immersion in the online world (31).

However, other studies have found that PIU can negatively predict self-control (21, 32). Ko et al. (33) found that college students with higher levels of PIU exhibited higher novelty seeking and risk-taking tendencies, as well as poorer self-control. Similarly, Reed et al. (34) through experiments discovered that individuals reporting higher levels of PIU exhibited greater impulsivity and lower levels of self-control when exposed to the internet. These research findings consistently indicate that self-control can also be an outcome variable of PIU.

The aforementioned arguments underscore the necessity for longitudinal investigations to explore the relationship between self-control and PIU. As cross-sectional data precludes testing directionality, we propose a longitudinal study utilizing a cross-lagged panel model to delineate predictive associations between internet self-control and PIU use over time.

### 1.2 Mediating role of meaning in life

Basic psychological needs theory posits that effective self-control enables adolescents to make self-directed choices aligned with internalized values, thereby fulfilling their core autonomy requirements (35). Eakman (36) states that meaningful activity fulfills basic psychological needs (i.e., autonomy, competence, and relatedness) and contributes to meaning in life. Conversely, those whose core needs remain unfulfilled are more likely to perceive their lives as lacking meaning. Meaning in Life refers to the sense made of, and significance felt regarding, the nature of one’s being and existence. This concept emphasizes the individual’s sense of purpose and values, highlighting their subjective sense of goals and the significance they attach to their own existence (37).

Research indicates that a high level of control is effective in promoting positive self-evaluation, goal-seeking, and positive actions, thereby facilitates a meaningful life for an individual (38). Conversely, low levels of control may lead individuals to develop negative emotions and withdrawal behaviors, and even to develop learned helplessness, which undermines the experience of meaning in life (39). That is, self-control is associated with successful progress toward goals and involves the ability to organize and structure one’s life in a way that may imbue it with coherence, comprehensibility and order. The benefits of self-control may extend beyond general life success and happiness, making a salient contribution to the meaningfulness of one’s life (40). Individuals who experience a greater sense of meaning in life tend to view their existence as meaningful and exhibit stronger self-control in managing undesirable emotions and behaviors (41). On the other hand, a prolonged absence of meaning in life might give rise to impulsive ideas taking momentary control, potentially leading to detrimental outcomes, such as addiction and criminal behavior (42).

Liu et al. (43) suggest that PIU arises as a consequence of unmet psychological needs in individuals, leading them to seek compensation through excessive internet use. Individuals who experience long-term deficits in their meaning in life often find their psychological needs unfulfilled in reality. As a result, they are more likely to engage in excessive internet use as a “pathological compensation,” ultimately leading to PIU (24). Students with a lower sense of meaning in life are more vulnerable to the impact of stress (44). Therefore, they may resort to addictive behaviors as a coping mechanism to escape from stress (25), such as PIU (16), alcohol consumption, smoking, and substance abuse (45–47). Research on mindfulness interventions based on existential therapy has also found that students who experience meaning in life through mindfulness meditation can reduce the severity of their PIU (48). Furthermore, excessive internet use has been found to have a detrimental effect on an individual’s sense of meaning in life (49). A bidirectional relationship may exist between meaning in life and PIU. Individuals with a low sense of meaning in life may seek refuge in the online world as a means of escaping the pressures of reality, which can lead to the development of PIU. Conversely, individuals who are extensively addicted to the internet may encounter difficulties in achieving personal accomplishments and fulfillment in real-life activities. Consequently, when detached from the virtual realm, they may experience a sense of inner emptiness and a lack of meaning in life. This, in turn, intensifies their PIU, giving rise to increasingly severe psychological problems (50).

Drawing on the basic psychological needs theory, prior research indicates that internet self-control fulfills core autonomy and competence demands, thereby enhancing one’s perceptions of meaning in life (51, 52). In turn, the sense of meaningfulness, arising from relatedness needs being met, serves to mitigate risks of PIU (53, 54). In addition, increased PIU can diminish meaning in life, further weakening internet self-control. Therefore, we hypothesized that meaning in life may act as a mediator in the relationship between internet self-control and PIU.

### 1.3 The present study

Based on the aforementioned literature, our study explores the relationship between self-control and PIU in the online environment. Taking a positive psychology perspective, our study aims to identify effective strategies for the prevention and intervention of PIU among Chinese adolescents. Therefore, our initial step involves utilizing a cross-lagged panel model with a 5-month time lag to examine the relationship between internet self-control and PIU. Subsequently, we constructed separate mediation models, with internet self-control and PIU as the independent variables, to explore the longitudinal relationships between the two constructs and the role of meaning in life as a mediator. Our first objective is to examine the relationship between internet self-control and PIU using a cross-lagged panel model with a 5-month time lag. Subsequently, we developed a mediation model to examine the longitudinal relationship between internet self-control and PIU, with a specific emphasis on the mediating role of meaning in life. Our second objective is to identify effective intervention strategies that can promote positive outcomes. To guide our study, we put forth two hypotheses:

*H1*: Internet self-control and PIU have a negative bidirectional predictive relationship.

*H1a*: Internet self-control at T1 negatively predicts PIU at T2.

*H1b*: PIU at T1 negatively predicts internet self-control at T2.

*H2*: Meaning in life mediates the relationship between internet self-control and PIU.

*H2a*: Meaning in life mediates the negative predictive effect of internet self-control at T1 on PIU at T2.

*H2b*: Meaning in life mediates the negative predictive effect of PIU at T1 on internet self-control at T2.

## 2 Materials and methods

### 2.1 Procedure and participants

This study has received approval from the Research Ethics Committee of Central China Normal University. Participants were recruited from two schools in the central region of China. All participants, along with their parents and teachers, were informed about the purpose of the study and signed informed consent forms. Participants were also informed that they had the right to withdraw from the study at any time without facing any negative consequences. After each measurement, participants received a monetary compensation of ¥5.

Employing a cluster sampling methodology, the current study employed classrooms as the sampling units. Prior studies employing cross-lagged investigation methodologies have recommended a temporal span of 4–6 months between assessments. In alignment with these findings and in consideration of the practical aspects of the assessment environment within the project context, this study has chosen to implement a 5-month interval between two consecutive rounds of assessments (55, 56). At the baseline (T1), a total of 850 questionnaires were administered, out of which 790 were completed, resulting in a response rate of 92.94%. For the follow-up assessment (T2), conducted 5 months later, 800 questionnaires were distributed, and 713 surveys were returned, yielding a response rate of 89.13%. No interventions took place between the two tests. After removing invalid responses, the final longitudinal sample consisted of 659 adolescents, including 331 males (50.23%) and 328 females (49.77%). Among them, 205 (31.11%) were in seventh grade, 187 (28.38%) were in eighth grade, 135 (20.49%) were in ninth grade, and 132 (20.03%) were in 10th grade. The average age of the participants was 13.61 years (SD = 1.409).

### 2.2 Measurement

#### 2.2.1 Internet self-control

Internet self-control was assessed in this study using a subscale from Adolescent Internet Adaptability Scale of Wang et al. (57). This subscale comprises five items, with an example item being “I will arrange my time on the Internet in a planned way.” Participants rated their agreement with each item on a six-point Likert scale, ranging from 1 (strongly disagree) to 6 (strongly agree). The Internet self-control subscale demonstrated strong internal consistency (T1α = 0.784, T2α = 0.783). The average score was then calculated for data analysis.

#### 2.2.2 Meaning in life

Meaning in life was evaluated using the 10-item Meaning in Life Questionnaire (58), adapted from the original scale developed by Steger (37). The Meaning in life questionnaire utilized a seven-point Likert scale, ranging from 1 (strongly disagree) to 7 (strongly agree). It consisted of two dimensions: presence of meaning and searching for meaning. An example item from the scale is “I am searching for the meaning of my life.” Higher scores on the scale indicated higher levels meaning in life. The Meaning in life questionnaire demonstrated strong internal consistency (T1α = 0.841, T2α = 0.872). The average score of Meaning in life was used for further analysis.

#### 2.2.3 Problematic internet use

Problematic internet use was assessed using the Chinese version of the self-rating Diagnostic Questionnaire of PIU questionnaire (C-IAD) (59), adapted from the PIU Disorder questionnaire developed by Young (5). The C-IAD utilizes a five-point Likert scale (1 = not at all, 5 = always) to measure the severity of PIU. An example item includes “Do you stay online longer than originally intended?” Higher scores on the scale indicate higher levels of PIU. This scale has also demonstrated strong internal consistency (T1α = 0.882, T2α = 0.918). The average score was used for data analysis.

### 2.3 Data analysis

The data entry process was conducted using Epi-data, and subsequent statistical analyses were performed after thorough error checking. Firstly, Welch’s *t*-tests in SPSS 25.0 were employed to compare the follow-up and attrition samples, with the aim of determining whether any significant differences existed between the two groups. Pearson’s correlation coefficient was then utilized to explore the associations and detect correlations between the main variables. Prior to conducting the mediation analyses, the data were checked to ensure they met the assumptions for mediation analysis. These include: linear relationships between variables, uncorrelated residuals, no multicollinearity, and univariate and multivariate normal distributions of the variables. Subsequently, a cross-lagged panel model was developed using Mplus 8.0 to investigate the predictive relationship between Internet self-control and PIU. Lastly, a longitudinal mediation model was constructed using Mplus 8.0 to examine the potential mediating role of meaning in life in the relationship between internet self-control and PIU.

## 3 Results

### 3.1 Attrition rate analysis

Welch’s *t*-test was employed to compare differences in the main variables between the follow-up sample and the attrition sample at T1 (60). The results, as presented in Table 1, indicated no significant differences between the two groups of participants on all key variables (*p* > 0.05). This indicated that the data were missing at random.

**Table 1:** Comparison of differences between the tracking and attrition samples (*M* ± *SD*).

Variables	Tracking samples (*n* = 617)	Attrition sample (*n* = 173)	*t*	*p*
Internet self-control	4.256 ± 1.202	4.486 ± 1.173	1.881	0.062
Meaning in life	5.003 ± 1.207	4.983 ± 1.141	−0.162	0.871
PIU	1.940 ± 0.848	2.092 ± 0.906	1.625	0.106

Welch’s *t*-test.

### 3.2 Correlation analysis

Table 2 presents the descriptive statistics and analysis of adolescent internet self-control, meaning in life, and PIU. Pearson correlations were conducted to examine the associations between these variables at baseline (T1) and the 5-month follow-up (T2). The results revealed significant negative correlations between internet self-control at both time points and PIU at T1 and T2. Additionally, significant positive correlations were found between internet self-control and meaning in life at T1 and T2. Furthermore, meaning in life at T1 and T2 exhibited significant negative correlations with PIU at T1 and T2.

**Table 2:** Descriptive statistics and correlations among variables.

	*M* ± *SD*	1	2	3	4	5	6
1. T1 ISC	4.338 ± 1.999	1					
2. T2 ISC	4.534 ± 1.097	0.366^**^	1				
3. T1 MIL	5.055 ± 1.209	0.278^**^	0.294^**^	1			
4. T2 MIL	4.929 ± 1.228	0.282^**^	0.460^**^	0.515^**^	1		
5. T1 PIU	1.966 ± 0.842	−0.342^**^	−0.212^**^	−0.232^**^	−0.219^**^	1	
6. T2 PIU	2.153 ± 0.995	−0.278^**^	−0.258^**^	−0.202^**^	−0.246^**^	0.569^**^	1

ISC is internet self-control, MIL is meaning in life, and PIU is problematic internet use; ^**^p < 0.01.

### 3.3 Cross-lagged panel analysis of internet self-control and PIU

Before the structural equation model analysis, we conducted a regression analysis. In this analysis, T1 PIU was set as the dependent variable, with T1 Internet self-control and T1 meaning in life as the independent variables. The results indicated a Variance Inflation Factor (VIF) of less than 10, suggesting that the variables exhibited linear relationships and met the assumptions of normality, homoscedasticity, and the absence of multicollinearity (see Appendix sections).

**Figure 1:**
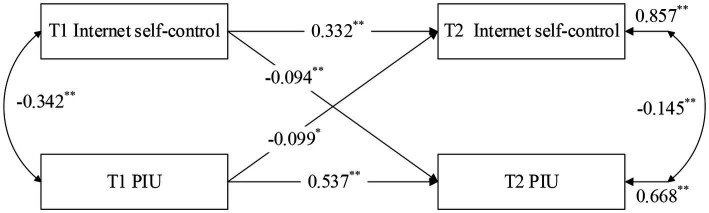
Cross-lagged panel model of internet self-control and PIU. ^**^p < 0.01, ^*^p < 0.05.

To examine the predictive relationships between internet self-control and PIU (H1a and H1b), a cross-lagged panel modeling was conducted using Mplus 8.0 (see Figure 1).

After controlling for baseline (T1) PIU, T1 internet self-control exhibited a significant negative prediction on PIU at the 5-month follow-up (T2) (β = −0.094, *p* < 0.01). Similarly, after controlling for T1 internet self-control, T1 PIU significantly negatively predicted T2 internet self-control (β = −0.099, *p* < 0.05). These results suggest a reciprocal predictive relationship between PIU and self-control. Thus, H1a and H1b are supported.

### 3.4 Longitudinal mediation model analysis of meaning in life

Cross-lagged panel modeling verified the bidirectional predictive relationship between internet self-control and PIU. To examine the mediating role of meaning in life, we conducted a Mediation Analysis using Mplus 8.0 and constructed two models. We tested the structural model to examine the proposed associations among variables. Indirect effects were calculated using bias-corrected bootstrapping (5,000 bootstrap samples) with 95% confidence intervals (CIs). In Mediation Analysis Model 1, we tested H2a with internet self-control as the independent variable and PIU as the dependent variable. In Mediation Analysis Model 2, we reversed the variables and tested H2b, with PIU as the independent variable and internet self-control as the dependent variable.

#### 3.4.1 Mediation analysis model 1: longitudinal effects of internet self-control on problematic internet use: the mediating role of meaning in life

**Figure 2:**
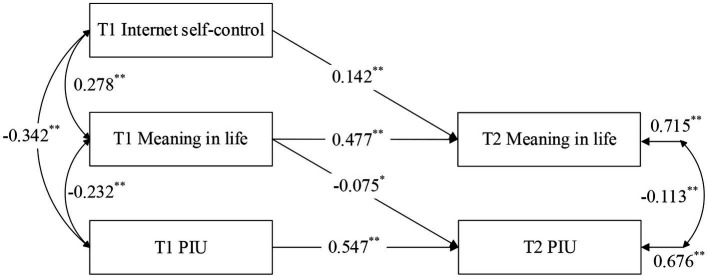
Longitudinal effects of internet self-control on PIU: the mediating role of meaning in life. ^**^p < 0.01, ^*^p < 0.05.

To test H2a, a mediation analysis was conducted to examine the longitudinal effects of internet self-control on PIU and the potential mediating role of meaning in life (see Figure 2: Mediation Analysis Model 1). The analysis provided satisfactory support for this hypothesis.

Results revealed that baseline (T1) internet self-control and T1 meaning in life significantly predicted meaning in life at the five-month follow-up (T2) (*β* = 0.142, *p* < 0.01; *β* = 0.477, *p* < 0.01). Furthermore, T1 meaning in life and T1 PIU significantly predicted T2 PIU (*β* = −0.075, *p* < 0.01; *β* = 0.547, *p* < 0.01). These findings suggest that higher levels of internet self-control are associated with higher levels of meaning in life, while higher levels of meaning in life are associated with lower levels of PIU.

When controlling for T1 meaning in life (as a covariate), T1 internet self-control continued to be a significant predictor of T2 meaning in life (*β* = 0.142, *p* < 0.01). Similarly, when controlling for T1 PIU, T1 meaning in life remained a significant predictor of T2 PIU (*β* = −0.075, *p* < 0.05). Model fit was assessed using established criteria of CFI and TLI values ≥0.9, RMSEA values ≤0.8, and SRMR values <0.05 for an acceptable fit (61). The model demonstrated strong fit (CFI = 0.985, TLI = 0.948, RMSEA = 0.075, SRMR = 0.023). In summary, the results suggest that meaning in life serves as a longitudinal mediator in the longitudinal effects of internet self-control on PIU. Thus, H2a was supported.

#### 3.4.2 Mediation analysis model 2: longitudinal effects of problematic internet use on internet self-control: the mediating role of meaning in life

**Figure 3:**
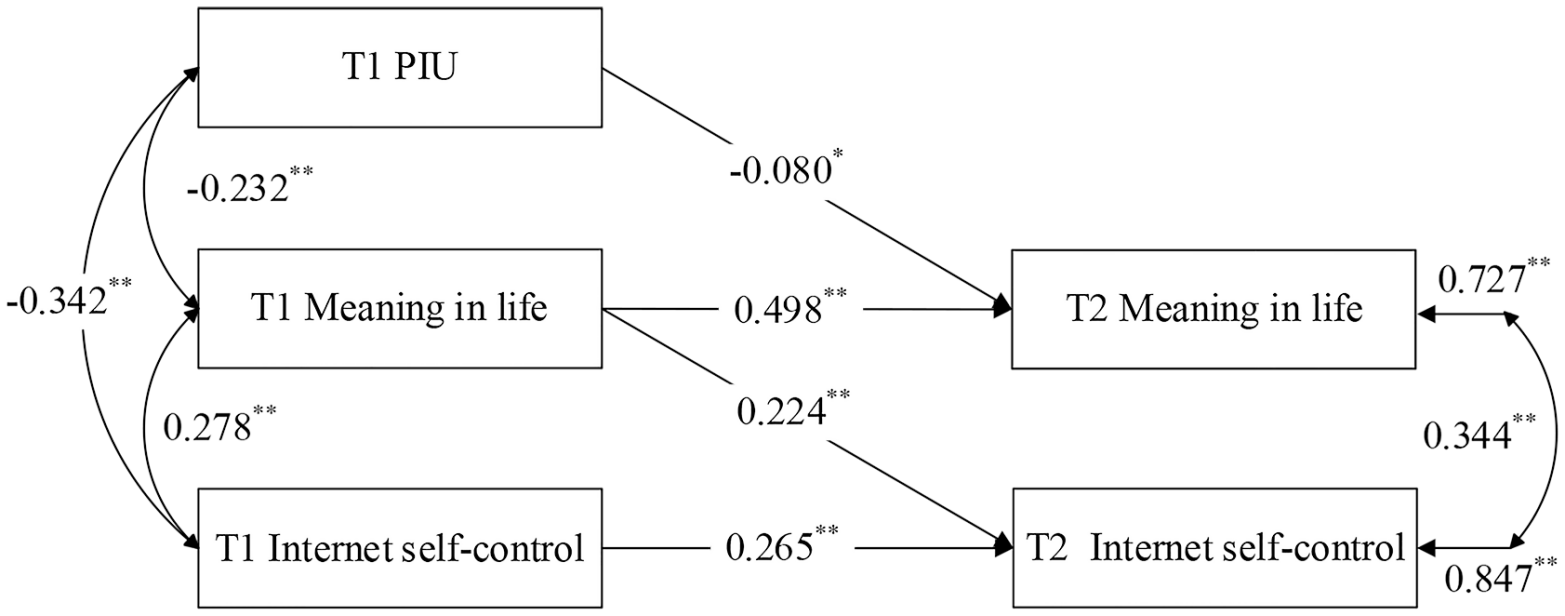
Mediating analysis model 2: Longitudinal effects of PIU on internet self-control: the mediating role of meaning in life. ^**^p < 0.01, ^*^p < 0.05.

To test H2b, mediation analysis was also conducted to examine the longitudinal effects of PIU on self-control, and the potential mediating role of meaning in life (see Figure 3: Mediation Analysis Model 2). However, this model demonstrated poor fit with the data and failed to provide satisfactory support for this hypothesis.

The results revealed that baseline (T1) PIU and T1 meaning in life significantly predicted meaning in life at the 5-month follow-up (T2) (*β* = −0.080, *p* < 0.05; *β* = 0.498, *p* < 0.01). Additionally, T1 meaning in life and T1 internet self-control significantly predicted T2 internet self-control (*β* = 0.224, *p* < 0.01; *β* = 0.265, *p* < 0.01). These findings suggest that higher levels of initial PIU are associated with lower levels of subsequent meaning in life, while higher levels of baseline meaning in life are linked to improved follow-up internet self-control.

When controlling for T1 meaning in life, T1 PIU still significantly predicted T2 meaning in life (*β* = −0.080, *p* < 0.05). Similarly, when controlling for T1 internet self-control, T1 meaning in life remained a significant predictor of T2 internet self-control (*β* = 0.224, *p* < 0.01). However, Model 2 demonstrated poor fit based on recommended criteria (61) (CFI = 0.963, TLI = 0.871, RMSEA = 0.110, SRMR = 0.035), with TLI < 0.9 and RMSEA > 0.8. In summary, meaning in life did not mediate the longitudinal effects of PIU on internet self-control. Thus, H2b was not supported.

The results of the two mediation analyses (Mediation Analysis Model 1 and 2) indicated that meaning in life mediated the longitudinal effect of internet self-control on PIU but did not mediate the longitudinal effect of PIU on internet self-control. Therefore, H2a is supported, while H2b is not supported.

## 4 Discussion

Adopting a positive psychology perspective, this two-wave longitudinal study aimed to elucidate the relationship and underlying mechanisms between internet self-control and PIU. Using cross-lagged panel models, the reciprocal predictive relationships between internet self-control and PIU were examined. Through longitudinal mediation models, the research elucidated the longitudinal mediating role of meaning in life within this association. This approach addressed limitations of cross-sectional studies and offered insights into the bidirectional relationships between self-control and PIU over time and broadening the research perspective on PIU beyond static assessments.

### 4.1 Bidirectional effects between self-control and PIU

The cross-lagged panel analysis demonstrated a reciprocal prediction relationship between internet self-control and PIU across timepoints.

Specifically, the results of the cross-lagged panel analysis indicated that baseline (T1) internet self-control significantly negatively predicted follow-up (T2) PIU, providing support for research H1a. This finding aligns with the basic psychological needs theory, which suggests that individuals with high levels of internet self-control are more capable of autonomously managing their online behaviors (27). They can effectively control their internet usage time and activities to avoid excessive immersion. Conversely, those with low internet self-control, particularly inexperienced adolescents, are more susceptible to becoming addicted to the short-term gratification offered by the open, anonymous, and virtual nature of the online world. They tend to struggle to exert self-control and effectively regulate their intended usage, ultimately leading to PIU (29). In the same vain, previous research has shown that modifying specific cognitions and expectations related to internet use can enhance internet self-control and reduce levels of PIU (62).

Moreover, T1 PIU was found to significantly negatively predict T2 internet self-control, indicating that higher initial levels of PIU undermine subsequent self-control capabilities, supporting research H1b. This finding is consistent with previous studies, as one of the diagnostic criteria for PIU is “unsuccessful attempts to control” or “loss of control” (28, 63). This criterion is also commonly included in questionnaires assessing PIU (5). Consequently, individuals with higher scores of PIU often exhibit characteristics of impaired online control, such as an inability to regulate the time and frequency of their internet use and becoming deeply engrossed in the online world, finding it difficult to detach themselves.

Overall, the results reveal bidirectional effects, whereby internet self-control mitigates later PIU severity, while PIU in turn erodes self-control resources. This reciprocal causation highlights the important interplay between self-control capacities and problematic internet use. Building upon this insight, this study introduces meaning in life as a mediating variable to further explore the underlying mechanisms between these two constructs.

### 4.2 Meaning in life mediates internet self-control and PIU

Meaning in life is an important protective factor against PIU (64). However, the current body of research has not adequately addressed the role of meaning in life in the reciprocal relationship between internet self-control and PIU. In contributing to the literature, our study demonstrates that “meaning in life” serves as a crucial mediating factor between internet self-control and PIU over time. In other words, internet self-control influences adolescents’ PIU both directly and indirectly through meaning in life, supporting H2a.

This finding is consistent with the basic psychological needs theory proposed by Ryan and Deci (65). According to this theory, individuals with higher levels of internet self-control are more likely to experience a sense of autonomy and competence, enabling them to engage in meaningful activities and pursue personal goals. As a result, they develop a stronger sense of purpose and meaning in life. This increased meaning in life provides individuals with a sense of fulfillment in their everyday lives, reducing their inclination toward PIU.

Therefore, our results suggest that reducing an individual’s level of PIU can be achieved not only by directly improving internet self-control but also indirectly by enhancing their sense of meaning in life. These research findings highlight the significance of interventions in PIU that go beyond cultivating individuals’ internet self-control abilities. It is also important to focus on enhancing their meaning in life. By encouraging individuals to pursue fulfillment and achieve goals in real-life activities, it is possible to reduce their reliance on the internet and mitigate the risk of addiction.

Furthermore, our research revealed that meaning in life does not mediate the impact of PIU on internet self-control. Our second hypothesis (H2b) did not find empirical support. The impact of PIU on reducing self-control may occur through direct mechanisms (e.g., reduced inhibitory control or impaired cognitive functioning) (32). Therefore, PIU may directly undermine self-control abilities without being mediated by meaning in life. Moreover, unmeasured factors like depression, anxiety, stress, or social support might mediate the relationship between PIU and self-control. Therefore, the null finding in our study highlights the complexity of the relationships between PIU, self-control, and meaning in life, suggesting the possibility of other moderating factors or bidirectional relationships.

### 4.3 Limitations and future research directions

While this was grounded in the perspective of positive psychology and employed a longitudinal research design, offering insights into the underlying mechanisms of the internal role of internet self-control on PIU, it is not without limitations.

Firstly, the reciprocal predictive relationship between internet self-control and PIU was examined using longitudinal data, which has inherent constraints. For instance, while this study examined mediation effects using a 5-month interval, which is informed by the established literature, future research is recommended to conduct measurements over varied lag periods (e.g., 3, 6, and 9 months, etc.) to validate our results and gain nuanced insights into how these relationships evolve over time. Secondly, we opted for path analysis over structural equation modeling (SEM) in our study due to our specific research design and sample size. Future studies could consider employing SEM for a more nuanced understanding of the relationships between variables we examined in this study. Lastly, the study primarily focused on a sample of Chinese middle and high school students, with a relatively small sample size. Future research should aim to expand the sample size to encompass diverse populations in different regions.

### 4.4 Significance

Our study addresses the ongoing debates surrounding self-control and PIU in the online context and holds significant implications. Theoretically, investigating the longitudinal relationship between internet self-control and PIU helps reveal the nature of their relationship and underlying mechanisms. It enriches the existing research on PIU from a positive psychology perspective. Moreover, while meaning in life did not mediate the effect of PIU on self-control in this study, further research is warranted to understand the mechanisms linking PIU and self-control. The null finding provides opportunities to develop more nuanced models of these relationships in future studies.

From a practical standpoint, our research provides empirical evidence and data support for interventions targeting PIU. Specifically, it highlights the importance of internet self-control and meaning in life in fostering healthy internet use habits among adolescents. Firstly, internet self-control directly influences adolescents’ internet usage behavior. Adolescents with higher levels of internet self-control are better able to regulate their online time and behaviors, resist online temptations, and maintain moderate usage. This helps prevent excessive immersion on the internet and reduces the risk of PIU. Secondly, meaning in life plays a mediating role between adolescents’ internet self-control and PIU. For adolescents with lower levels of internet self-control, enhancing their meaning in life can guide them in contemplating and exploring their life goals, values, and interests. It helps them recognize the role of the internet in achieving these goals, enabling them to use the internet purposefully and avoid the risks of PIU.

Therefore, by delving into the mechanisms through which internet self-control and meaning in life impact adolescent PIU, we can develop targeted intervention strategies and methods. This offers support to adolescents already ensnared by PIU.

## 5 Conclusion

Utilizing a two-wave longitudinal design and a sample of Chinese adolescents, this study revealed the reciprocal predictive relationships between internet self-control and PIU through cross-lagged modeling. Specifically, internet self-control at baseline predicted subsequent PIU, and baseline PIU also predicted subsequent internet self-control. The longitudinal mediation model demonstrated that meaning in life mediated the longitudinal effect of internet self-control on PIU, but not the effect of PIU on self-control. These findings suggest the feasibility of reducing adolescent PIU levels directly or indirectly by improving internet self-control and meaning in life. The study provides empirical evidence and potential pathways for PIU interventions from a positive psychology perspective.

## Data availability statement

The raw data supporting the conclusions of this article will be made available by the authors, without undue reservation.

## Ethics statement

The studies involving humans were approved by Research Ethics Committee of Central China Normal University. The studies were conducted in accordance with the local legislation and institutional requirements. Written informed consent for participation in this study was provided by the participants’ legal guardians/next of kin.

## Author contributions

WW: Conceptualization, Funding acquisition, Investigation, Resources, Supervision, Writing – review & editing. JY: Data curation, Formal analysis, Project administration, Writing – original draft, Writing – review & editing. YZ: Formal analysis, Visualization, Writing – original draft. DH: Investigation, Methodology, Writing – review & editing. XZ: Funding acquisition, Supervision, Validation, Writing – review & editing.
