# Supplementary material for: Longitudinal relationship between internet self-control and problematic internet use among Chinese adolescents: mediating role of meaning in life
Source: Front Psychiatry. 2023 Dec 7;14:1258673. doi: 10.3389/fpsyt.2023.1258673 (PMC10748819; doi:10.3389/fpsyt.2023.1258673)
Supplement: Supplementary file 1 [file Data_Sheet_1.pdf]

Appendix Sections

Normal distribution analysis

The skewness and kurtosis tests of each variable are as follows:

Table 1 skewness and kurtosisi test

	Skewness	Kurtosis
T1 ISC	-0.353	-0.465
T2 ISC	-0.500	-0.066
T1 MIL	-0.290	-0.108
T2 MIL	-0.027	-0.033
T1 PIU	1.110	1.300
T2 PIU	0.841	0.103

Note: ISC is internet self-control, MIL is meaning in life, PIU is Problematic internet use

The results show that skewness and kurtosis were less than ±2, this means that all variables follow the normal distribution.

Regression analysis

The results show that VIF were less than 10, this means that there is no multicollinearity between the variables.

Table 2 Regression analysis

	B	*t*	*VIF*
T1 ISC	-0.211	-7.958**	1.084
T1 MIL	-0.104	-3.955**	1.084

**p<0.01

The results of residual correlation test are shown in Figure 1

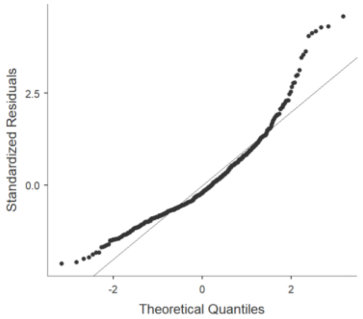

Figure1 Q-Q Plot

The Q-Q Plot shows no significant correlation between the residuals.

Multivariate normal distribution analysis

The multivariate normal distribution analysis are as follows:

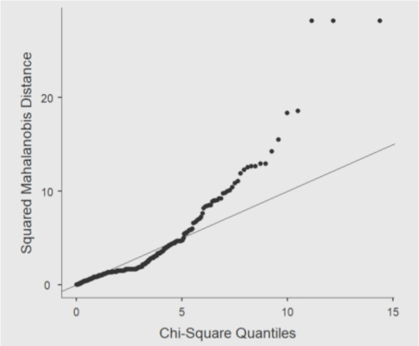

Figure 2 P-P Plot

Among them, the independent variable includes:

T1 internet self-control, T1 meaning in life, T2 internet self-control and T2 meaning in life.

The dependent variable includes:

T1 problematic internet use and T2 problematic internet use.

Figure 2 means that the data basically satisfy the multivariate normal distribution.
